# Supplementary material for: c-MYC is Transcribed in a Circadian Manner and Acts a Clock Disruptor whose Timing Minimizes its Impacts
Source: bioRxiv. 2026 May 29:2026.05.26.727929. Preprint. [Version 1] doi: 10.64898/2026.05.26.727929 (PMC13232071; doi:10.64898/2026.05.26.727929)
Supplement: 1 — S1 Fig. Sequence analysis of the c-MYC promoter-reporter. Shown is a graphical representation of (above) and sequence information for (below) the c-MYC:luc promoter, highlighting important regulatory elements relative to the P1 start site: an E-box (shown in red) and the nuclease hypersensitive element III (1) (NHEIII1; underlined). S2 Fig. Luciferase assay validation of U2OS-c-MYC:luc cells. The data shown for each condition represent the average of three biological replicates (N = 3), with the error bars indicating the standard deviation. A student’s t-test was performed to calculate the statistical significance of the biological replicates for each cell line (*** p < 0.001). S3 Fig. Individual raw and detrended bioluminescent traces for U2OS-c-MYC:luc reporter cell lines. Shown are raw traces excluding the first 24 h (left), and traces after detrending by removing the average of a 24-h sliding window (right). (N=17 with 9 outliers in gray for c-MYC:luc, where N is the number of replicates). S4 Fig. Raw and detrended bioluminescent traces for mBmal1 and BMAL1 reporter cell lines. Shown are raw traces excluding the first 24 h (left), and traces after detrending by removing the average of a 24-h sliding window (right). For both raw and detrended data, the mean is plotted as a solid line, with the standard error of the mean shown as a semi-transparent envelope around the mean. (N=3 for mBmal1:luc and N=12 for hBMAL1:luc, where N = number of data sets or replicates). S5 Fig. Period and phase offset values for mBmal1:luc and BMAL1:luc cells. The period (above) and phase offset (below) values were determined by fitting a damped cosine curve to the detrended mBmal1:luc and hBMAL1:luc traces. (N=3 for mBmal1:luc and N=12 for hBMAL1:luc, where N = number of data sets or biological replicates). S6 Fig. Additional circadian parameter analysis for PER2:luc, BMAL1:luc, and c-MYC:luc reporter cells. The peak-to-peak period (A) was estimated by averaging the differences in timin [file NIHPP2026.05.26.727929V1-supplement-1.pdf]

bioRxiv preprint doi: https://doi.org/10.64898/2026.05.26.727929; this version posted May 29, 2026. The copyright holder for this preprint (which was not certified by peer review) is the author/funder, who has granted bioRxiv a license to display the preprint in perpetuity. It is made available under a CC-BY 4.0 International license.

c-MYC:luc

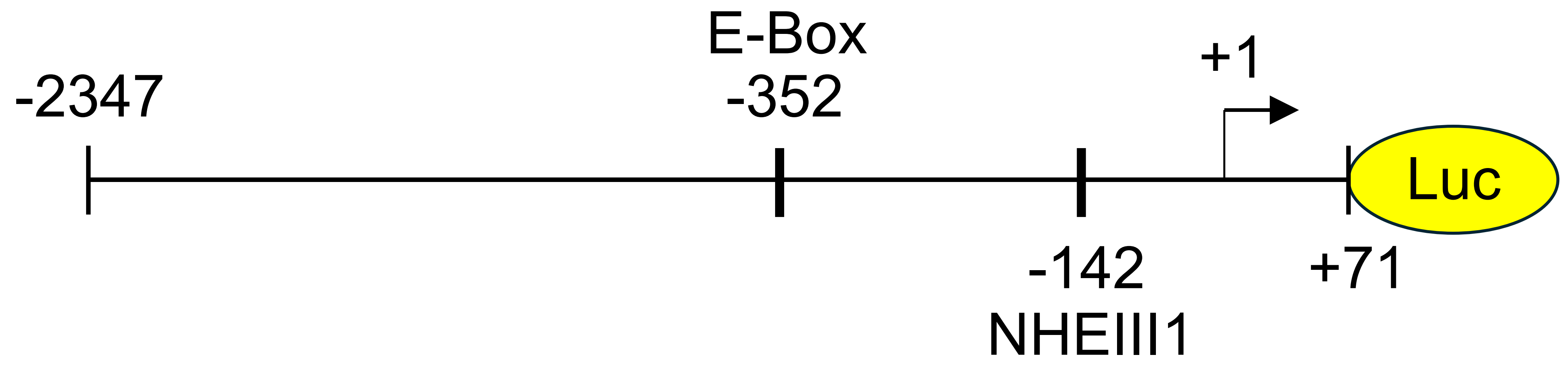

E-Box

-385 GGTGGGCGCG CAGTGCGTTC TCGGTGTGGA GGG**CAGCTG**T TCCGCCTGCG ATGATTTATA CTCACAGGAC -316

-315 AAGGATGCGG TTTGTCAAAC AGTACTGCTA CGGAGGAGCA GCAGAGAAAG GGAGAGGGTT TGAGAGGGAG -246

-245 CAAAAGAAAA TGGTAGGCGC GCGTAGTTAA TTCATGCGGC TCTCTTACTC TGTTTACATC CTAGAGCTAG -176

NHEIII1

-175 AGTGCTCGGC TGCCCGGCTG AGTCTCCTCC CCA**CCTTCCC CACCCTCCCC ACCCTCCCCA** TAAGCGCCCC -106

-105 TCCCGGGTTC CCAAAGCAGA GGGCGTGGGG GAAAAGAAAA AAGATCCTCT CTCGCTAATC TCCGCCCACC -36

P1

-35 GGCCCTTTAT AATGCGAGGG TCTGGACGGC TGAGG**A**CCCC CGAGCTGTGC TGCTCGCGGC CGCCACCGCC +35

bioRxiv preprint doi: https://doi.org/10.64898/2026.05.26.727929; this version posted May 29, 2026. The copyright holder for this preprint (which was not certified by peer review) is the author/funder, who has granted bioRxiv a license to display the preprint in perpetuity. It is made available under a CC-BY 4.0 International license.

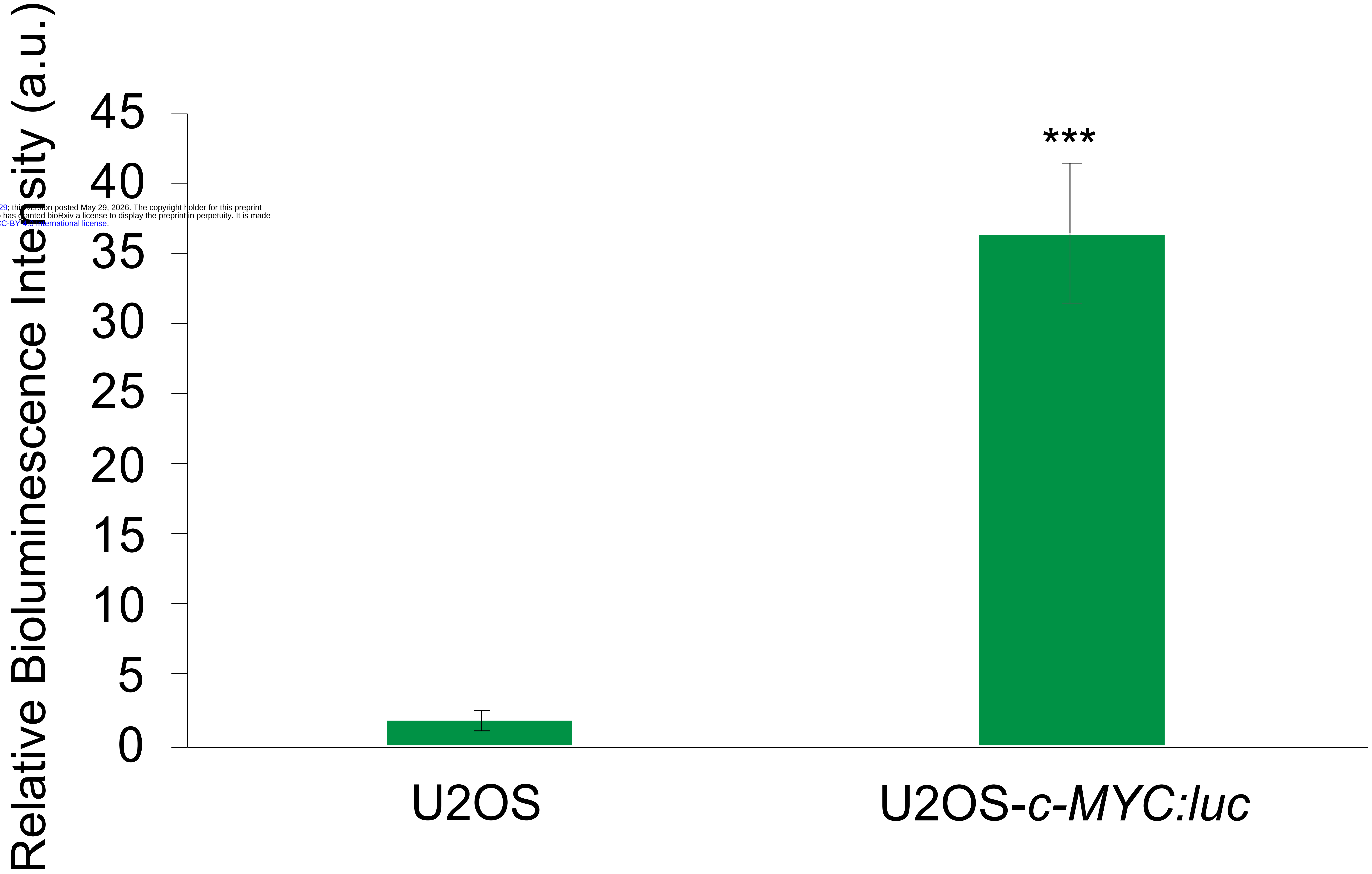

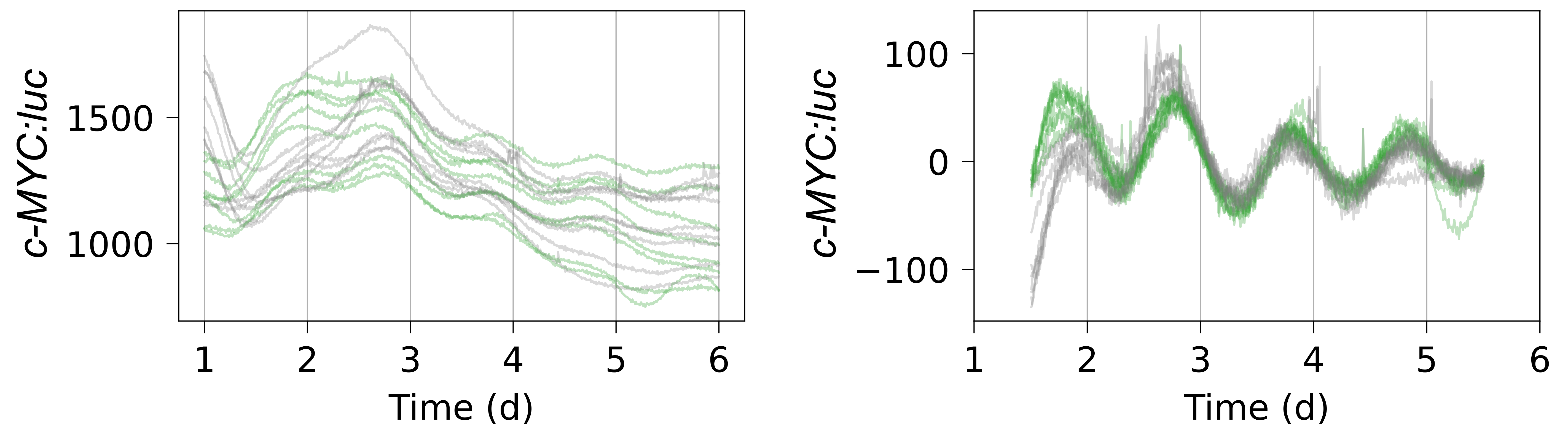

c-MYC:luc
1500
1000
1
2
3
4
5
6
Time (d)
c-MYC:luc
100
0
−100
1
2
3
4
5
6
Time (d)

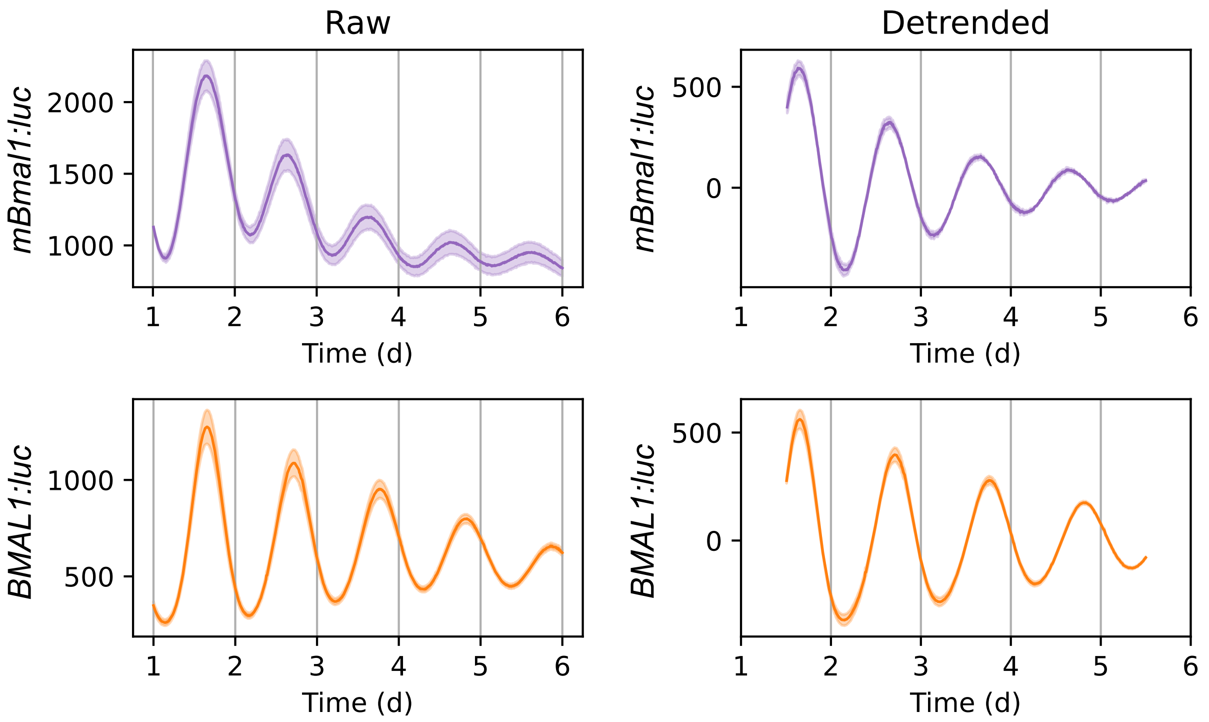
Raw
Detrended
mBmal1:luc
2000
1500
1000
500
0
BMAL1:luc
1000
500
500
0
1
2
3
4
5
6
Time (d)

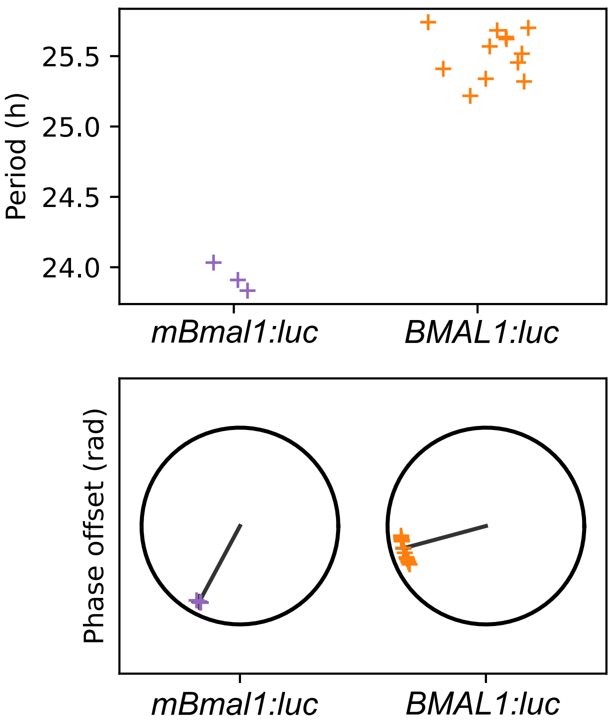
25.5
25.0
24.5
24.0
Period (h)
mBmal1:luc
BMAL1:luc
Phase offset (rad)
mBmal1:luc
BMAL1:luc

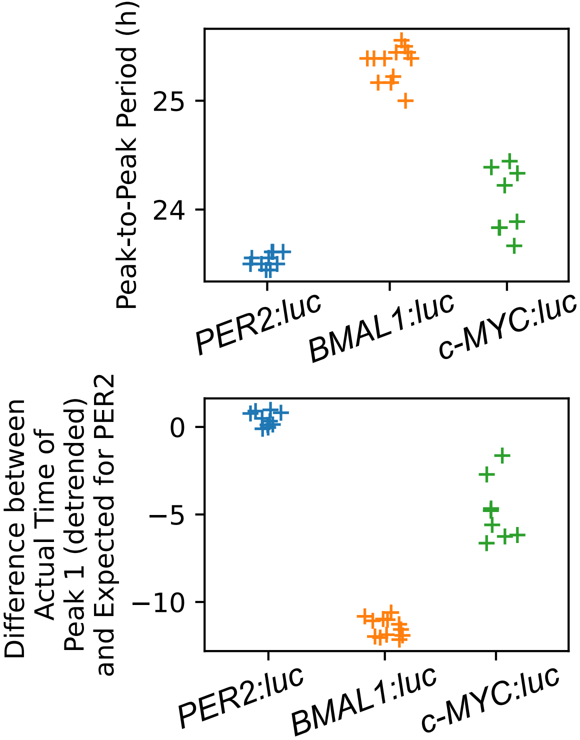

Peak-to-Peak Period (h)
25
24
PER2:luc
BMAL1:luc
c-MYC:luc
Difference between
Actual Time of
Peak 1 (detrended)
and Expected for PER2
0
−5
−10
PER2:luc
BMAL1:luc
c-MYC:luc
